# Supplementary material for: Comprehensive nucleosome mapping of the human genome in cancer progression
Source: Oncotarget. 2015 Dec 31;7(12):13429–45. doi: 10.18632/oncotarget.6811 (PMC4924652; doi:10.18632/oncotarget.6811)
Supplement: Supplementary file 1 [file oncotarget-07-13429-s001.pdf]

Comprehensive nucleosome mapping of the human genome in cancer progression

Supplementary Material

Contents

I. Table 1 page 2

II. Table 2 page 3

III. Table 3 page 4

IV. Figure 1 page 5-6

V. Figure 2 page 7

I. Table 1 Patient samples, MNase preparation and sequencing processing. Lung and colon adenocarcinoma patients, matched normal and tumor, from whom the samples used in the sequencing study were taken.

Sample ID	MNase Preparation	Sequencing Processing
LAC 4137, N and T	identical MNase prep samples from Druliner et. al., 2013; new digestion from remaining nuclei	original prep on HiSeq2000 (1 lane, multiplexed) and MiSeq, new prep on HiSeq2000 (1 lane, multiplexed); all reads combined for analyses
LAC 1357, N and T	identical MNase prep samples from Druliner et. al., 2013	sequenced on MiSeq (4 lanes, multiplexed)
LAC 873, N and T	new MNase digestion from remaining nuclei (original nuclei isolation from Druliner et. al., 2013)	sequenced on HiSeq (1 lane, multiplexed)
LAC 386, N and T	new MNase digestion from remaining nuclei (original nuclei isolation from Druliner et. al., 2013)	sequenced on HiSeq (1 lane, multiplexed)
CRC 512, N and T	tissue obtained 2013, nuclei isolated, cross-linked, MNase digested as described in Druliner, et. al., 2013 (patients previously unpublished)	sequenced on HiSeq (1 lane, multiplexed)
CRC 524, N and T	tissue obtained 2013, nuclei isolated, cross-linked, MNase digested as described in Druliner, et. al., 2013	sequenced on HiSeq (1 lane, multiplexed)
CRC 533, N and T	tissue obtained 2013, nuclei isolated, cross-linked, MNase digested as described in Druliner, et. al., 2013	sequenced on HiSeq (1 lane, multiplexed)

II. Table 2 Primers for enrichment qPCR. Primer design for performing qPCR to determine enrichment of on-target regions and depletion of off-target regions.

Gene Name	Genome Region	On-target/ Off-target	Primer Name (forward)	Primer Sequence (forward)	Primer Name (reverse)	Primer Sequence (reverse)
ATM	chr11:108093855-108094255	On-target	ATM_ONTARGET_F	CTGGGAAATTCAGTCGTGTG	ATM_ONTARGET_R	TATTGGCCAAGTCCGCTAAG
ATM	chr11:108098504-108098615	Off-target	ATM_OFFTARGET_F	AATTTAAGCGCCTGATTCGAG	ATM_OFFTARGET_R	CCTTGTTTGGAATCTGAATGC
RHOC	chr1:113250099-113250499	On-target	RHOC_ONTARGET_F	AGATGTCCACCCTCTTGTTCC	RHOC_ONTARGET_R	CCAGGGAAGAAAGCGAATTG
RHOC	chr1:113246266-113246422	Off-target	RHOC_OFFTARGET_F	TTGCTGAAGACGATGAGGAG	RHOC_OFFTARGET_R	CAATCCGAAAGAAGCTGGTG
ITGA4	chr2:182321015-182321415	On-target	ITGA4_ONTARGET_F	TATGGCTGTCTCTCTGGTTGC	ITGA4_ONTARGET_R	AACGCAACACACCTGAACTG
ITGA4	chr2:182322923-182323044	Off-target	ITGA4_OFFTARGET_F	CAACGCTTCAGTGATCAATCC	ITGA4_OFFTARGET_R	GAGCTGTTCGCACGTCTG

III. Table 3 Read data for LAC samples following sequencing. The data corresponding to the targeted regions in every case made up over 90% of the total sequencing reads.

Sample	Raw	Aligned to genome	On-Target (reads aligned to seqcap oligos)	Off-Target (reads not aligned to seqcap oligos)	% On-Target	% Off-Target
4137N	46,300,390	37,216,362	33,674,285	3,542,077	0.90	0.10
4137T	38,324,430	29,778,407	26,702,281	3,126,126	0.90	0.10
1357N	9,026,754	8,101,614	7,332,706	768,908	0.91	0.09
1357T	6,790,830	5,653,261	5,104,155	549,106	0.90	0.10
873N	14,454,359	12,675,104	11,661,232	1,013,872	0.92	0.08
873T	19,465,767	17,205,831	15,856,972	1,348,859	0.92	0.08
386N	24,910,145	22,121,267	20,416,785	1,704,482	0.92	0.08
386T	25,054,967	22,300,403	20,623,027	1,677,376	0.92	0.08

IV. Figure 1

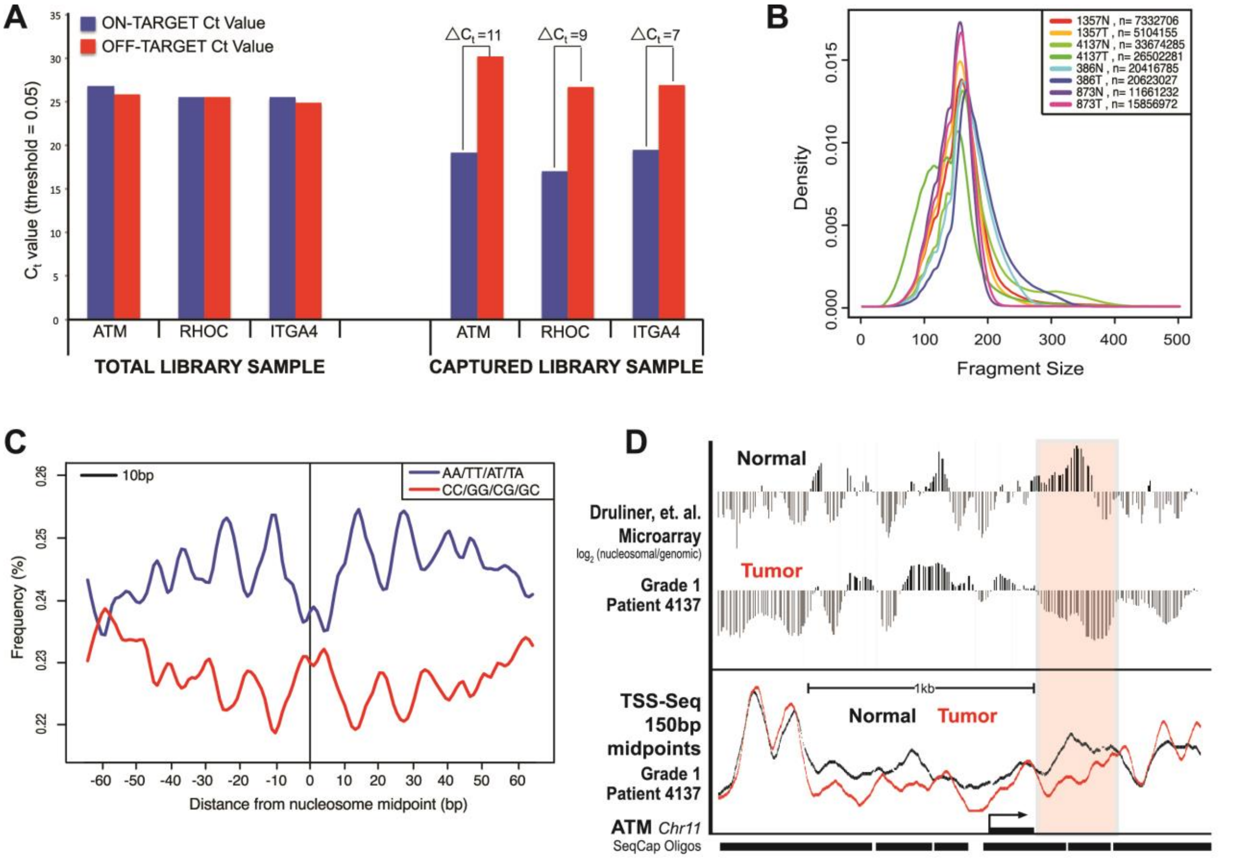

mTSS-seq quality control, dinucleotide frequency and concordance with previous microarray work. (A) qPCR following mTSS-seq shows ~1:500 enrichment for the target regions (2kb surrounding TSS) compared to the off-target regions (outside the 2kb surrounding the TSS). Selected genes; ATM, RHOC and ITGA4 for both the pre-capture pooled total library and the sequence captured pooled library are shown, Ct values are on the y-axis. (B) Distribution of mTSS-seq fragment sizes following sequencing, determined after mapping to the human genome. The fragments for all samples (n= 8 genomes: 4patients, matched normal-tumor) peak at ~150bp, as expected for MNase protected DNA. Fragment sizes were inferred from the separation of adaptors after paired-end sequencing. (C) Nucleosomal dinucleotide composition shown for LAC patient #4137 Normal for fragments of sizes 151-152bp, with a 130bp window being shown. The x-axis is the distance from the nucleosome midpoint (center indicated by solid black vertical line). The blue line is the A/T containing dinucleotide frequency and the red line is the G/C containing dinucleotide frequency. (D) Nucleosome distribution maps from our previously published microarray analysis in these patients show identical regions of change to those determined by mTSS-seq. Frequency distributions of midpoint data for LAC patient #4137 Normal (top panel in microarray section; and black line in mTSS-seq section) and Tumor (bottom panel in microarray section; and red line in mTSS-seq section) were plotted in the UCSC genome browser with respect to the 2kb surrounding the transcription start site of the

ATM gene (http://genome.ucsc.edu). The y-axis indicates the number of aligned reads. The track marked ‘Microarray’ shows intensity values for MNase digested mononucleosome DNA compared to bare genomic DNA, hybridized to a tiling array taken from the data set described in Druliner et al., 2013, Cell Cycle. Positive peaks in the array track are indicate the presence of positioned nucleosomes and are co-incident with 150-bp midpoints. The region of change is highlighted by a shaded red box is similar between microarray and sequencing data.

V. Figure 2

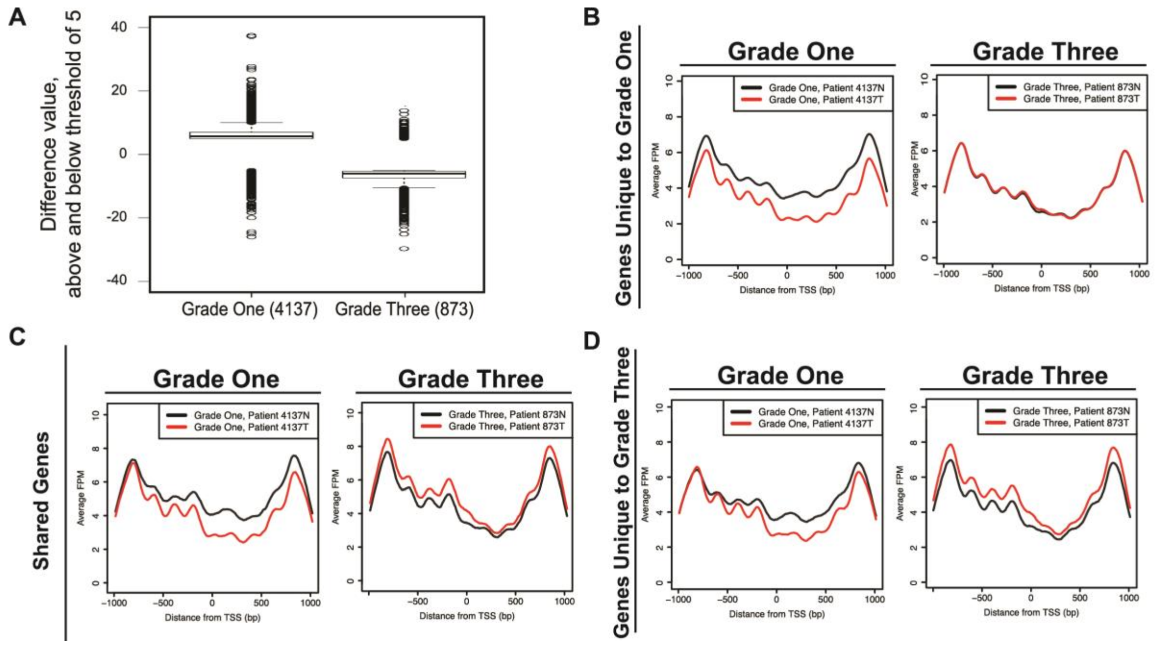

Low grade patients show a greater degree of difference than the grade three patients. (A) The threshold applied to determine regions of difference was the most stringent cut-off that discriminated between the samples, while revealing a substantial enough number of regions to perform downstream analyses in the grade three patients since there were far less regions of change than in the grade one patients. Boxplots for all the difference values for grade one and grade three patients above and below the -5-5 threshold are shown. Overall, the total difference values for the grade one patients have a much higher range than the values for grade three patients, showing that the grade one patients are more different overall than the grade three patients. We plotted the average nucleosome distribution averages for normal (black lines) and tumor (red lines) for genes in all categories shown in Figure 5.7. We confirmed that for all the genes in each category (B) unique to grade one, (C) shared, and (D) unique to grade three (corresponding to 9,864, 2,008, and 2,825 genes, respectively) the average nucleosome distribution plots showed more change in the grade one patients than in the grade three patients. We found that in all cases the grade one patients showed a much greater degree of difference than the grade three patients.
